# Potential contribution of the neurodegenerative disorders risk loci to cognitive performance in an elderly male gout population

**DOI:** 10.1097/MD.0000000000008195

**Published:** 2017-09-29

**Authors:** Lin Han, Zhaotong Jia, Chunwei Cao, Zhen Liu, Fuqiang Liu, Lin Wang, Wei Ren, Mingxia Sun, Baoping Wang, Changgui Li, Li Chen

**Affiliations:** a Department of Endocrinology Qilu Hospital of Shandong University, Jinan; b Gout Laboratory, The Affiliated Hospital of Qingdao University, Qingdao; c State Key Laboratory of Stem Cell and Reproductive Biology, Institute of Zoology, Chinese Academy of Sciences, Beijing, China.

**Keywords:** cognitive function, genome-wide association study, gout, neurodegenerative disorders risk loci, single-nucleotide polymorphism

## Abstract

Cognitive impairment has been described in elderly subjects with high normal concentrations of serum uric acid. However, it remains unclear if gout confers an increased poorer cognition than those in individuals with asymptomatic hyperuricemia. The present study aimed at evaluating cognitive function in patients suffering from gout in an elderly male population, and further investigating the genetic contributions to the risk of cognitive function.

This study examined the cognitive function as assessed by Mini-Mental State Examination (MMSE) and the Montreal Cognitive Assessment (MoCA) in 205 male gout patients and 204 controls. The genetic basis of these cognitive measures was evaluated by genome-wide association study (GWAS) data in 102 male gout patients. Furthermore, 7 loci associated with cognition in GWAS were studied for correlation with gout in 1179 male gout patients and 1848 healthy male controls.

Compared with controls, gout patients had significantly lower MoCA scores [22.78 ± 3.01 vs 23.42 ± 2.95, *P* = .023, adjusted by age, body mass index (BMI), education, and emotional disorder]. GWAS revealed 7 single-nucleotide polymorphisms (SNPs) associations with MoCA test at a level of conventional genome-wide significance (*P* < 9.6 × 10^–8^). The most significant association was observed between rs12895072 and rs12434554 within the *KTN1* gene (*P*_adjusted_ = 4.2 × 10^−9^, *P*_adjusted_ = 4.7 × 10^–9^) at 14q22. The next best signal was in *RELN* gene (rs155333, *P*_adjusted_ = 1.3 × 10^–8^) at 7q22, while the other variants at rs17458357 (*P*_adjusted_ = 3.98 × 10^–8^), rs2572683 (*P*_adjusted_ = 8.9 × 10^–8^), rs12555895 (*P*_adjusted_ = 2.6 × 10^–8^), and rs3764030 (*P*_adjusted_ = 9.4 × 10^–8^) were also statistically significant. The 7 SNPs were not associated with gout in further analysis (all *P* > .05).

Elderly male subjects with gout exhibit accelerated decline in cognition performance. Several neurodegenerative disorders risk loci were identified for genetic contributors to cognitive performance in our Chinese elderly male gout population. Larger prospective studies of the cognitive performance and genetic analysis in gout subjects are recommended.

## Abstract 2

Supplemental Digital Content is available in the text

## 1 Introduction

The potential role of uric acid (UA) in cognitive function has been an area of interest. On the one hand, UA is a major natural antioxidant in human plasma. Elevated UA levels have been hypothesized to protect against oxidative damage in several neurodegenerative disorders.^[1–5]^ On the other hand, for each molecule of UA produced, the enzymatic degradation of xanthine simultaneously produces superoxide anions, which are among the most powerful pro-oxidants.^[6]^ Recently, a series of cross-sectional observational studies showed that high normal concentrations of serum uric acid (SUA) are associated with mild cognitive dysfunction (MCD), especially among those aged 60 years or older. In a cross-sectional observational study, Schretlen et al^[7]^ found that elderly subjects with higher baseline SUA are 2.7 to 5.9 times more likely to score in the bottom quartile on measures of cognitive outcomes. They also proposed that severity of cerebral ischemia might mediate the association between SUA and cognitive dysfunction.^[8]^ This finding was replicated in another 814 older persons of the population-based Rotterdam Study and showed that higher SUA_base_ correlated with greater white matter atrophy.^[9]^ The latter studies indicated that the relationship between higher SUA and cognitive dysfunction may be mediated by white matter atrophy and cerebral ischemia.^[10]^

It is known that gender-specific differences in the effects of SUA on the cognition. A cross-sectional study in a cohort of 694 unrelated Chinese aged 90 to 108 years showed that only among men, the higher level of SUA is related to the risk of cognitive impairment.^[11]^ Similarly, another subcohort study of patients with chronic cardiovascular disease showed that SUA is a predictor on the domain of attention in particular among older men.^[12]^

The results of the genetic analyses are also equivocal. A recent study with 343 Parkinson disease patients reported that no significant association was detected between genetic variants related to SUA levels and the risk of neurocognitive disorder due to Parkinson disease,^[13]^ although Houlihan et al^[14]^ found a genetic mechanism behind a possible association between higher SUA concentrations and better cognitive performance in later life.

In gout, the combination of persistent systemic and joint inflammation and hyperuricemia may accelerate cognitive decline. Deposition of urate crystal material in vessel walls has been proposed to cause neutrophil and platelet activation, while less-differentiated monocytes produce abundant amounts of inflammatory mediators along with chemokines and adhesion molecules that promote cardiovascular damage.^[15–17]^ Furthermore, comparing patients with asymptomatic hyperuricemia, gout patients are associated with higher SUA, longer duration, and more likely to be receiving drug treatment. Therefore, the confusion with regard to gout role in cognitive performance is also enhanced by the complication of the disease. However, it remains unclear whether gout confers an increased poorer cognition than those in individuals with asymptomatic hyperuricemia.

The goal of this study, therefore, was to examine the association between gout and cognitive function using case–control data first, and then performed genetic analysis with GWAS to determine the genetic contributions to the risk of cognitive function in an elderly male gout population.

## 2 Materials and methods

### 2.1 Study design and Study population

Flowchart of study design is presented in Supplementary Figure 1. We recruited a total of 205 male gout patients and 204 male gout-free controls for the cognitive assessments from December 2013 to August 2015, which were older than 60 years. The genetic basis of cognitive measures was evaluated by association analysis with genome-wide SNP data in 102 male gout patients. Furthermore, 7 loci associated with cognition in GWAS were studied for correlation with gout in 1179 male gout patients and 1848 healthy male controls.

All samples were unrelated Han Chinese individuals recruited from the affiliated Hospital of the Qingdao Medical College, in the coastal city of Qingdao in Shandong province, China. All the gout patients were diagnosed by a clinical endocrine physician according to the criteria from the American College of Rheumatology in 1977.^[18]^ Likewise, normal male controls without a personal or familial history of hyperuricemia or gout or other serious illness were collected. All subjects with a history of neurological disease (e.g., large vessel stroke, Parkinson disease, Alzheimer disease, severe brain injury, seizure, multiple sclerosis, brain infection, meningitis) were excluded.

This study was approved by the Ethics Committee of the Affiliated Hospital, Qingdao University, and conducted in accordance with the ethical guidelines of the 1975 Declaration of Helsinki. All subjects gave written informed consent.

### 2.2 Demographic and clinical assessment

The demographic and clinical characteristics of the subjects, including age, gender, years of education, relevant history regarding gout, and its treatment, were collected by face-to-face interviews at the baseline evaluation. Body mass index (BMI) was calculated as weight (kg)/height (m^2^). Current SUA and before urate-lowering treatment of SUA were measured using an automated multichannel chemistry analyzer (Model 200; Toshiba, Tokyo, Japan).

### 2.3 Cognitive function and emotional disorder assessment

Two hundred five male gout patients and 204 male gout-free controls underwent the following tests: the MMSE and the MoCA to evaluate the cognitive impairment, and the hospital anxiety and depression scale (HADS) to assess the depressive mood. All the interviews were done face to face by nurses or researcher who had attended unified training several times before. All the tests were carried out according to provided guidelines and procedures.

MMSE is extensively used as a screening test for dementia and MMSE score of <18 is used to define subjects with moderate/severe cognitive impairment. The MMSE gives 24 out of 30 points for memory, language, and orientation and only 1 out of 30 for visuoconstructive function and is insensitive to MCD.^[19–22]^

MoCA is a brief screening tool for cognitive impairment. It is a 1-page protocol that assesses 8 cognitive domains (attention, executive functions, calculations, language, working memory and recall, abstraction, orientation, and visuospatial processing), including a Clock Drawing Test and an oral Verbal Fluency Test.^[23]^ Many studies of brain function have shown that the MoCA is superior to the MMSE in detecting MCD, showing good sensitivity and very good specificity.^[24,25]^

In this study, all subjects were tested with MMSE at the start of the assessment to exclude moderate/severe cognitive impairment, and no patient and control had a score < 18. Therefore, MoCA was administered to 205 gout patients and 204 controls to further detect MCD.

HADS is a self-assessment scale and is designed to provide a screening device for anxiety and depression in a general hospital setting.^[26]^ The anxiety and depression subscales are valid measures of severity of emotional disorder: for each part, a score below 8 is in the normal range, 8 to 10 is borderline and above 10 indicates a probable disorder of the relevant mood. Epidemiological evidence strongly supports that mood is closely linked to cognitive function in elderly.

### 2.4 Genotyping

A 2-mL peripheral blood sample was collected from study participant into an EDTA tube. Genomic DNA was extracted using a whole-blood DNA isolation kit (Bioteke, Beijing, China). Samples were genotyped in the first stage of analysis using Axiom Genotyping Algorithm v1 (Axiom GT1).

For the sample filtering, arrays with generated genotypes for <95% of the loci were excluded. The heterozygosity rates were calculated and deviations of more than 6 s.d. from the mean were excluded. PLINK's identity by descent analysis was used to detect the hidden relatedness. When pairs of individuals had a PI_HAT > 0.25, the member of the pair with the lower call rate was excluded from the analysis; 102 gout patients were retained for further analyses. For the SNP filtering, SNPs with call rates < 95% in the samples were removed. SNPs with a minor allele frequency < 3% or SNPs that deviated significantly (*P* ≤ 1 × 10^–5^) from Hardy–Weinberg equilibrium were also excluded. A total of 518,904 SNPs passed the quality criteria and were used in the subsequent analyses.

Seven SNPs (rs17458357 at Chr2, rs2572683 at Chr3, rs155333 at Chr7, rs12555895 at Chr9, rs3764030 at Chr12, rs12895072 at Chr14, rs12434554 at Chr14) were analyzed by TaqMan technique. Direct sequencing of 5% randomly selected samples was applied to validate the genotyping assays. And, the success rate was 99.5%.

### 2.5 Statistical analysis

Demographic and clinical characteristics were summarized as means ± standard deviations and compared between the 2 groups. All variables were examined for normality of distribution using the Kolmogorov–Smirnov test, and data were considered normally distributed if *P* > .05. One-way analysis of variance (ANOVA) test was performed to find if differences are statistically significant when showing a normal distribution. Measurements that were not normally distributed were tested using Kruskal–Wallis ANOVA test. Quality control filtering and all genetic association analysis were conducted using a linear regression model implemented in PLINK^[27]^ adjusted by the age, BMI, and education years covariates. The most conservative method (Bonferroni multiple significance test correction) was used to address the problem of multiple comparisons, and the cutoff value for significance was set at *P* = .05/518904 = 9.64 × 10^–8^. A Manhattan plot of the -log_10_ (*P* values) was generated Barrett et al.^[28]^ Regional plots were generated using the online tool Locus Zoom 1.2 37 (http://csg.sph. umich.edu/locuszoom/). The software package SPSS 16.0 (SPSS, Inc., Chicago, IL) was used for the statistical analysis.

## 3 Results

### 3.1 Clinical features of the study population

There was no significant difference in age and education between the gout patients and the controls (*P* = .408, *P* = .063). Compared with controls, gout patients had significantly higher BMI, higher current UA, and higher before urate-lowering treatment UA (*P* = .024, *P* < .001, *P* < .001). Among the 205 gout patients, the average disease duration of gout is 9.56 years (0–44). One hundred twenty-seven (61.95%) patients received UA-lowering therapy (Table 1).

**Table 1:**
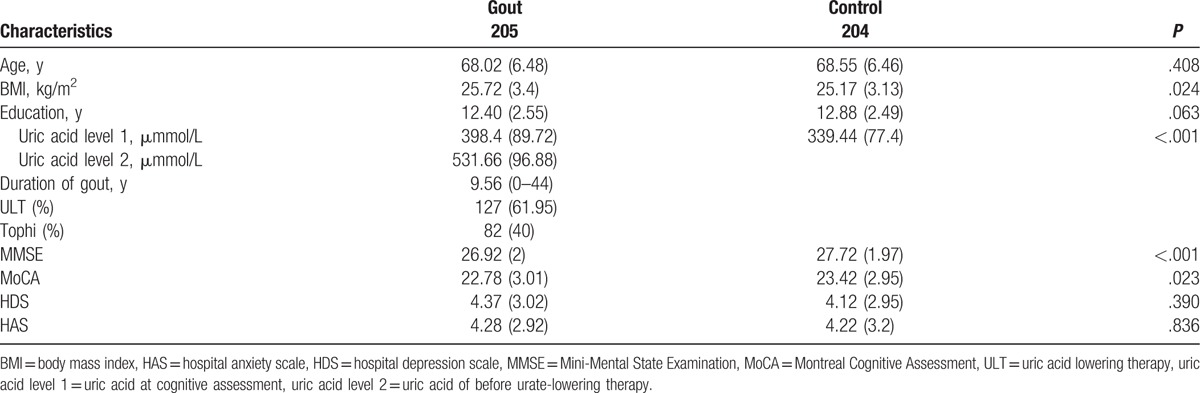
Demographic and clinical description of the analyzed sample.

### 3.2 Cognitive function and emotional disorder assessment

A total of 205 male gout patients and 204 male gout-free controls were recruited for the cognitive assessment. The decrease of the MMSE scores in the gout patients as compared with the controls was highly significant (*P* < .001, adjusted by age, BMI, education, and emotional disorder). The means of MMSE scores were 26.92 ± 2 for gout patients and 27.72 ± 1.97 for controls. No patient and control had a score <18 in this test; therefore, all subjects were retained for further analysis. The means of MoCA scores were 22.78 ± 3.01 for gout patients and 23.42 ± 2.95 for controls. MoCA score was significantly lower in patients with gout than in controls (*P* = .023, adjusted by age, BMI, education, and emotional disorder). Variations in HADS were not significantly different between the 2 groups (4.37 ± 3.02 vs 4.12 ± 42.95; *P* = .390 and 4.28 ± 2.92 vs 4.22 ± 43.20; *P* = .836). Data are presented in Table 1. One hundred two gout patients were selected by random from 205 gout patients for genetic analysis. The means of MoCA scores were not significantly different between 102 patients and 205 patients (22.78 ± 3.01 vs 23.15 ± 3.33; *P* = .836) (Supplementary Table 1).

### 3.3 Genetic association analysis of cognitive function

**Figure 1:**
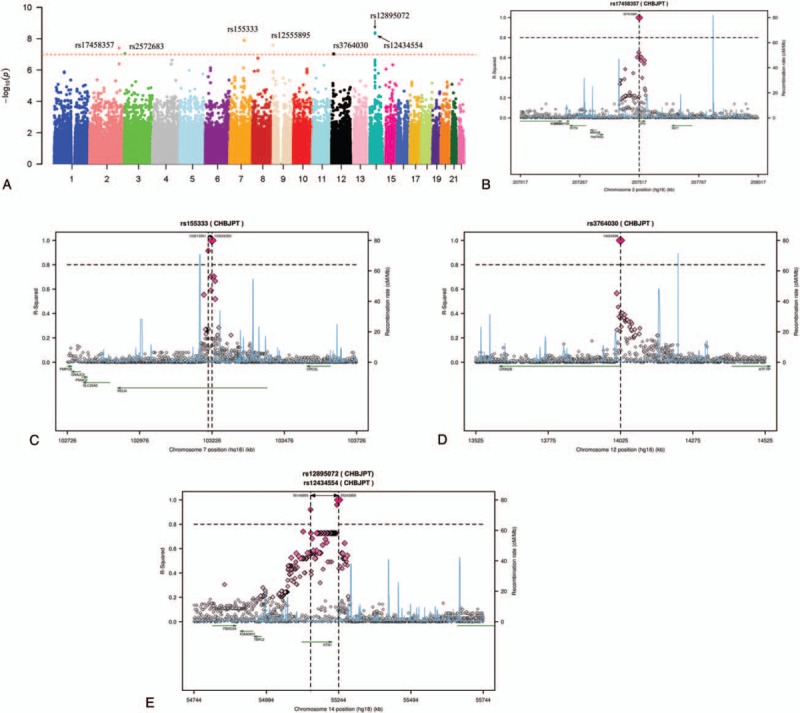
(A) The *P* value distribution for single-marker associations with MoCA test under an additive genetic model. (B) rs17458357 located in *CPO* gene. (C) rs155333 in *RELN* gene. (D) rs3764030 located in *GRIN2B* gene. (E) rs12895072 and rs12434554 within the *KTN1*gene.

Figure 1A shows the *P*-value distribution for single-marker associations with MoCA test under an additive genetic model adjusted for age at cognitive assessment, BMI, and education in 102 male cases across the whole genome. GWAS revealed 7 SNP associations with MoCA test at a level of conventional genome-wide significance (*P* < 9.6 × 10^–8^) (Table 2). Five top markers were mapped to 4 known genes, including carboxypeptidase O (*CPO*) (Fig. 1B), reelin (*RELN*) (Fig. 1C), glutamate receptor 2B subunit (*GRIN2B*) (Fig. 1D), and kinectin (*KTN1*) (Fig. 1E). The remaining 2 SNPs were not mapped to any gene regions. The most significant association was observed between rs12895072 and rs12434554 within the *KTN1* gene (*P*_adjusted_ = 4.2 × 10^–9^, *P*_adjusted_ = 4.7 × 10^–9^) at 14q22. The next best signal was in *RELN* gene (rs155333, *P*_adjusted_ = 1.3 × 10^–8^) at 7q22, while the other variants at rs17458357 (*P*_adjusted_ = 3.98 × 10^–8^), rs2572683 (*P*_adjusted_ = 8.9 × 10^–8^), rs12555895 (*P*_adjusted_ = 2.6 × 10^–8^), and rs3764030 (*P*_adjusted_ = 9.4 × 10^–8^) were also statistically significant (Table 2). To be noted, several SNPs within *KTN1* showed borderline associations with cognitive function in our study.

**Table 2:**
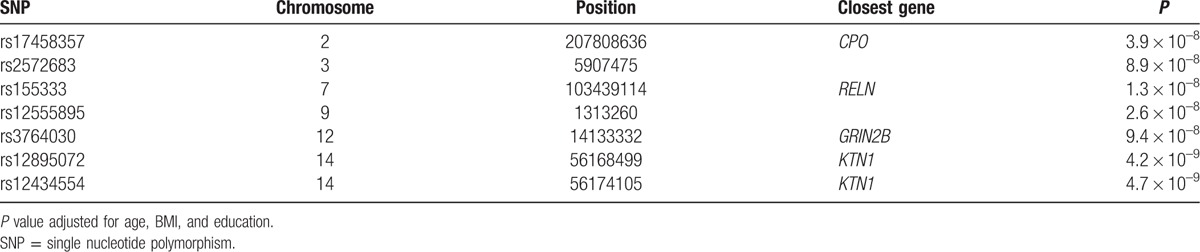
Statistically significant associations (assuming an additive model of inheritance) between SNPs and measure of MoCA.

Given the observed association between cognitive performance and the 7 top SNPs, we sought to further characterize this relationship. We evaluated the effects of demographic and clinical characteristics (age, BMI, education, duration of gout, tophi, UA) on cognitive performance. In linear regressions, no significant association was observed for the 7 top SNPs effect on cognitive function and gout characteristics or severity (all *P* > .05) (Supplementary Table 2).

### 3.4 Association of 7 top SNPs with the susceptibility to gout

To further investigate the association between the 7 top SNPs and gout, we have also genotyped the 7 top SNPs in a population of 1179 gout patients and 1848 controls. However, no SNP reached significance (all *P* > .05) (Table 3). Age is a well-known risk factor in the development and prognosis of cognitive decline; therefore, we further evaluated the genotype and allele frequencies in different age subgroups. For the individuals older than 60, the frequencies of 7 top SNPs were not significant between cases and controls (all *P* > .05). Moreover, we performed a stratified analysis according to the age of onset; none of the SNPs was found to be significantly associated with an increased risk of early onset (<30 years) compared with late onset (>60 years) (all *P* > .05) (Table 3).

**Table 3:**
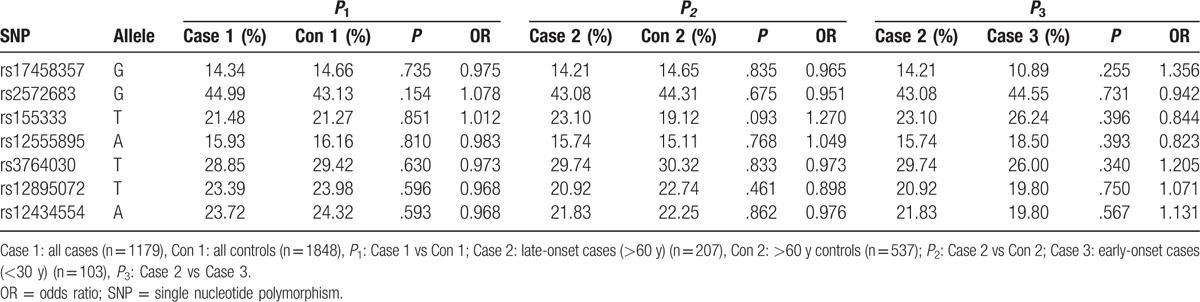
Association of 7 loci correlated with cognition in GWAS with the susceptibility to gout.

## 4 Discussion

The present study revealed that elderly male subjects with gout exhibit accelerated decline in cognition performance. We conducted a GWAS that revealed 7 SNPs associations with MoCA test at a level of conventional genome-wide significance (*P* < 9.6 × 10^–8^). To our knowledge, this is the first report that supports contribution of genetic variants to cognitive function among older male gout patients, showing that neurodegenerative risk variants are relevant to this phenotype among gout individuals.

The most significant association with cognitive decline was within the *KTN1* gene containing the SNP rs12895072 and SNP rs12434554 (*P*_adjusted_ = 4.2 × 10^–9^, *P*_adjusted_ = 4.7 × 10^–9^) at 14q22. The result is consistent with the finding of the published human brain structure GWAS, which implicated mainly volumes of the putamen and caudate nucleus.^[29]^*KTN1* encodes the protein kinectin, which is primarily found in the endoplasmic reticulum in the dendrites and thesoma of neurons.^[30]^ It plays a critical role in the regulation of neuronal cell shape, spreading, and migration through kinectin–kinesin interactions.^[31]^ In this study, other than the 2 SNPs, several SNP_s_ within *KTN1* showed borderline associations with cognitive function, which implicates that this gene plays a central role in gout patients’ cognitive function.

Second, we identified an intronic locus within *Reelin* (rs155333; 7q22.1; *P*_adjusted_ = 1.3 × 10^–8^) at 7q22. Reelin is an extracellular protein that is mainly involved in nerve cell migration, appropriate brain lamination, and synapse formation in the central nervous system.^[32]^ Many studies have reported that *reelin* gene SNPs are associated with depressive disorder, autism, schizophrenia, bipolar disorder, and lissencephaly.^[33–37]^ Function studies showed that the failure to regulate the *RELN* expression caused changes in the synaptogenesis and neural migration and the structural development of the brain regions related with nerve activity, attention, and impulsivity.^[38]^ Our results also showed that the gene was associated with cognitive decline.

The third strongest association with cognitive function was within the *CPO* gene containing the SNP rs17458357 (*P*_adjusted_ = 3.9 × 10^–8^). *CPO* is a previously uncharacterized and unique member of the *CPA* subfamily, which is highly expressed in intestinal epithelial cells in both zebrafish and human.^[39]^ Lyons and Fricker^[40]^ found that *CPO* is present on the brush border of the ileal mucosa where it is responsible for producing free glutamate and aspartate from dietary proteins. Dietary glutamate has been proposed to be the most important source of oxidative energy for intestinal enterocytes, revealing a critical role for CPO in the intestine.^[40,41]^ Abnormal intestinal oxidative stress has been noted as being a likely cause of gastrointestinal permeability, which can result in neuroinflammation and cognitive decline.^[42]^

The fourth strongest association with cognitive impairment was a SNP upstream of the *GRIN2B* gene (rs3764030; 12p12; *P*_adjusted_ = 9.4 × 10^–8^), which encodes the protein GluN2B, a receptor that is primarily expressed in the fronto-parieto-temporal cortex and is involved in learning and memory.^[43]^ Genetic variations within *GRIN2B* have been associated with cognitive phenotypes.^[44]^

These heritability estimates are in line with previous estimates in relatively healthy aging populations and suggest that several neurodegenerative disorders risk loci are important to cognitive performance in people with gout.

The links between UA and neurodegenerative disorders have long been reported in several studies.^[1,2,5]^ Consequently, cognitive decline and UA or gout may share common genetic factors. In fact, Houlihan et al^[14]^ tested *SLC2A9* SNPs, previously associated with UA levels, in ∼1000 Scots: the Lothian Birth Cohort 1936 (LBC1936). Four common SNP_s_ within *SLC2A9* were found to be associated with a general memory factor in LBC1936, while these associations were not replicated in Edinburgh Type 2 Diabetes Study.^[14]^ Similarly, a case–control study that included 95 Lewy body disorders patients and 76 controls performed an analysis of 3 SNPs in *SLC17A3* gene known to be associated with altered serum UA levels. The study concluded that SNP rs1165205 (*SLC17A3*) was weakly associated with altered cerebrospinal fluid UA levels^[45]^ and cerebrospinal fluid UA levels seem to be critically involved in neurodegenerative processes. In this study, we further investigate whether genome-wide significant loci of cognitive function are associated with the risk of gout. Unexpectedly, no single cognitive performance risk loci were associated with gout. Genetic risk for gout is often due to multiple SNPs in various locations on the chromosome with small individual effects; therefore, minor effects of SNPs and insufficient sample size are possible explanations for the nonreplication.

There are several limitations to this study. Sample size used in this study was still limited, especially for discovery sample sizes. This will be addressed in the future. Furthermore, cross-sectional design of the study does not allow for any prospective conclusion on the relationship of genetic variants. Finally, we do not detect rare genetic variants or copy-number variations by resequencing genes. As whole-genome sequencing becomes more affordable, such studies are expected to become feasible.

Despite these limitations, the present study has unique strengths, first, our sample benefits from the high homogeneity (Chinese Han from Shandong Province, China) and accuracy of the gout diagnosis. Second, patient data regarding emotional disorders that contribute by themselves to cognitive decline and disease information regarding gout characteristics or severity were collected. It is crucial to take them into account (based on multiple measurements, representing the course of gout better than a single risk factor measurement) in the statistical analysis.

To conclude, our study suggests that elderly male subjects with gout exhibit accelerated decline in cognition performance. Genetic analysis revealed a number of neurodegenerative disorders risk loci that warrant further consideration. Such identification is critical to understanding new pathways to prevent and treat this insidious complication of this increasingly prevalent disease. Larger prospective studies of the cognitive performance and genetic analysis in gout subjects are recommended.

## Supplementary Material

Supplemental Digital Content
