# Supplementary material for: Potential contribution of the neurodegenerative disorders risk loci to cognitive performance in an elderly male gout population
Source: Medicine (Baltimore). 2017 Sep 29;96(39):e8195. doi: 10.1097/MD.0000000000008195 (PMC5626325; doi:10.1097/MD.0000000000008195)
Supplement: Supplemental Digital Content [file medi-96-e8195-s001.doc]

Potential contribution of the neurodegenerative disorders risk loci to cognitive performance in [an elderly male gout population](http://www.sciencedirect.com/science/article/pii/S0140673699021959)

Lin Han et al.

Cognitive function and emotional disorder assessment

Gout patients (n=205)

Controls (n=204)

Investigation the genetic contributions to the risk of cognitive function in gout population

Gout patients (n=102)

Investigation the association between the

7 top-SNPs and gout

Gout patients (n=1179)

Controls (n=1848)

Supplementary Figure 1: Flowchart of study design.

Potential contribution of the neurodegenerative disorders risk loci to cognitive performance in [an elderly male gout population](http://www.sciencedirect.com/science/article/pii/S0140673699021959)

Lin Han et al.

Table S1. Montreal cognitive assessment (MoCA) mean scores for the gout patients.

|  | Gout 1 | Gout 2 | *P*-valve |
| --- | --- | --- | --- |
|  | 205 | 102 |  |
| MoCA | 22.78(3.01) | 23.15(3.33) | 0.237 |

Notes: MoCA: Montreal cognitive assessment; Gout 1: 205 male gout patients for cognitive assessments; Gout 2: 102 male gout patients for Genome-Wide Association Study.

Potential contribution of the neurodegenerative disorders risk loci to cognitive performance in [an elderly male gout population](http://www.sciencedirect.com/science/article/pii/S0140673699021959)

Lin Han et al.

Table S2. Linear regression analysis for top-SNP_S_ effect on cognitive function across proxies of gout characteristics or severity

| CHR | SNP |  | BETA | L95 | U95 | *P*-value |
| --- | --- | --- | --- | --- | --- | --- |
| 2 | rs17458357 | age | -0.114 | -0.176 | -0.052 | <0.001 |
|  |  | BMI | -0.096 | -0.288 | 0.095 | 0.330 |
|  |  | education  (years) | 0.814 | 0.187 | 1.441 | 0.013 |
|  |  | duration of gout (years) | -0.0189 | -0.0862 | 0.049 | 0.584 |
|  |  | tophi | 0.845 | -0.218 | 1.908 | 0.123 |
|  |  | uric acid (μmmol/L) | 0.005 | -0.001 | 0.011 | 0.089 |
| 3 | rs2572683 | age | -0.149 | -0.215 | -0.082 | <0.001 |
|  |  | BMI | -0.150 | -0.356 | 0.056 | 0.156 |
|  |  | education  (years) | 1.039 | 0.358 | 1.721 | 0.004 |
|  |  | duration of gout (years) | -0.024 | -0.097 | 0.049 | 0.516 |
|  |  | tophi | 0.873 | -0.299 | 2.044 | 0.148 |
|  |  | uric acid (μmmol/L) | 0.002 | -0.004 | 0.008 | 0.467 |
| 7 | rs155333 | age | -0.142 | -0.211 | -0.074 | <0.001 |
|  |  | BMI | -0.178 | -0.393 | 0.038 | 0.110 |
|  |  | education  (years) | 0.947 | 0.233 | 1.661 | 0.011 |
|  |  | duration of gout (years) | -0.039 | -0.115 | 0.037 | 0.321 |
|  |  | tophi | 1.278 | 0.096 | 2.46 | 0.037 |
|  |  | uric acid (μmmol/L) | 0.003 | -0.004 | 0.009 | 0.418 |
| 9 | rs12555895 | age | -0.146 | -0.212 | -0.079 | <0.001 |
|  |  | BMI | -0.164 | -0.369 | 0.042 | 0.123 |
|  |  | education  (years) | 0.993 | 0.31 | 1.677 | 0.005 |
|  |  | duration of gout (years) | -0.026 | -0.099 | 0.047 | 0.491 |
|  |  | tophi | 1.005 | -0.154 | 2.164 | 0.093 |
|  |  | uric acid (μmmol/L) | 0.001 | -0.005 | 0.007 | 0.702 |
| 12 | rs3764030 | age | -0.122 | -0.193 | -0.051 | 0.001 |
|  |  | BMI | -0.11 | -0.324 | 0.104 | 0.316 |
|  |  | education  (years) | 0.838 | 0.136 | 1.539 | 0.021 |
|  |  | duration of gout (years) | -0.039 | -0.113 | 0.035 | 0.305 |
|  |  | tophi | 1.283 | 0.124 | 2.441 | 0.033 |
|  |  | uric acid (μmmol/L) | 0.003 | -0.003 | 0.010 | 0.297 |
| 14 | rs12895072 | age | -0.123 | -0.185 | -0.61 | <0.001 |
|  |  | BMI | -0.164 | -0.355 | 0.026 | 0.095 |
|  |  | education  (years) | 0.906 | 0.276 | 1.537 | 0.006 |
|  |  | duration of gout (years) | -0.021 | -0.089 | 0.047 | 0.547 |
|  |  | tophi | 0.946 | -0.119 | 2.012 | 0.085 |
|  |  | uric acid (μmmol/L) | 0.003 | -0.003 | 0.008 | 0.361 |
| 14 | rs12434554 | age | -0.125 | -0.188 | -0.063 | <0.001 |
|  |  | BMI | -0.163 | -0.356 | 0.030 | 0.102 |
|  |  | education  (years) | 0.906 | 0.267 | 1.544 | 0.007 |
|  |  | duration of gout (years) | -0.021 | -0.09 | 0.048 | 0.55 |
|  |  | tophi | 1.005 | -0.073 | 2.083 | 0.071 |
|  |  | uric acid (μmmol/L) | 0.002 | -0.003 | 0.008 | 0.410 |

Notes: BMI: body mass index.
